# Replication, pathogenicity, and transmission of SARS-CoV-2 in minks

**DOI:** 10.1093/nsr/nwaa291

**Published:** 2020-12-08

**Authors:** Lei Shuai, Gongxun Zhong, Quan Yuan, Zhiyuan Wen, Chong Wang, Xijun He, Renqiang Liu, Jinliang Wang, Qinjian Zhao, Yuxiu Liu, Ningning Huo, Junhua Deng, Jingjing Bai, Hongchao Wu, Yuntao Guan, Jianzhong Shi, Kegong Tian, Ningshao Xia, Hualan Chen, Zhigao Bu

**Affiliations:** State Key Laboratory of Veterinary Biotechnology, Harbin Veterinary Researched Institute, Chinese Academy of Agricultural Sciences, Harbin 150069, People's Republic of China; National High Containment Laboratory for Animal Diseases Control and Prevention, Harbin 150069, People's Republic of China; State Key Laboratory of Veterinary Biotechnology, Harbin Veterinary Researched Institute, Chinese Academy of Agricultural Sciences, Harbin 150069, People's Republic of China; National High Containment Laboratory for Animal Diseases Control and Prevention, Harbin 150069, People's Republic of China; State Key Laboratory of Molecular Vaccinology and Molecular Diagnostics, National Institute of Diagnostics and Vaccine Development in Infectious Diseases, School of Public Health, Xiamen University, Xiamen 361102, People's Republic of China; State Key Laboratory of Veterinary Biotechnology, Harbin Veterinary Researched Institute, Chinese Academy of Agricultural Sciences, Harbin 150069, People's Republic of China; National High Containment Laboratory for Animal Diseases Control and Prevention, Harbin 150069, People's Republic of China; State Key Laboratory of Veterinary Biotechnology, Harbin Veterinary Researched Institute, Chinese Academy of Agricultural Sciences, Harbin 150069, People's Republic of China; State Key Laboratory of Veterinary Biotechnology, Harbin Veterinary Researched Institute, Chinese Academy of Agricultural Sciences, Harbin 150069, People's Republic of China; National High Containment Laboratory for Animal Diseases Control and Prevention, Harbin 150069, People's Republic of China; State Key Laboratory of Veterinary Biotechnology, Harbin Veterinary Researched Institute, Chinese Academy of Agricultural Sciences, Harbin 150069, People's Republic of China; National High Containment Laboratory for Animal Diseases Control and Prevention, Harbin 150069, People's Republic of China; State Key Laboratory of Veterinary Biotechnology, Harbin Veterinary Researched Institute, Chinese Academy of Agricultural Sciences, Harbin 150069, People's Republic of China; National High Containment Laboratory for Animal Diseases Control and Prevention, Harbin 150069, People's Republic of China; State Key Laboratory of Molecular Vaccinology and Molecular Diagnostics, National Institute of Diagnostics and Vaccine Development in Infectious Diseases, School of Public Health, Xiamen University, Xiamen 361102, People's Republic of China; National Research Center for Veterinary Medicine, No. 3 Cuiwei Road, High-Tech District, Luoyang 471003, People's Republic of China; National Research Center for Veterinary Medicine, No. 3 Cuiwei Road, High-Tech District, Luoyang 471003, People's Republic of China; National Research Center for Veterinary Medicine, No. 3 Cuiwei Road, High-Tech District, Luoyang 471003, People's Republic of China; National Research Center for Veterinary Medicine, No. 3 Cuiwei Road, High-Tech District, Luoyang 471003, People's Republic of China; National Research Center for Veterinary Medicine, No. 3 Cuiwei Road, High-Tech District, Luoyang 471003, People's Republic of China; National High Containment Laboratory for Animal Diseases Control and Prevention, Harbin 150069, People's Republic of China; State Key Laboratory of Veterinary Biotechnology, Harbin Veterinary Researched Institute, Chinese Academy of Agricultural Sciences, Harbin 150069, People's Republic of China; National Research Center for Veterinary Medicine, No. 3 Cuiwei Road, High-Tech District, Luoyang 471003, People's Republic of China; State Key Laboratory of Molecular Vaccinology and Molecular Diagnostics, National Institute of Diagnostics and Vaccine Development in Infectious Diseases, School of Public Health, Xiamen University, Xiamen 361102, People's Republic of China; State Key Laboratory of Veterinary Biotechnology, Harbin Veterinary Researched Institute, Chinese Academy of Agricultural Sciences, Harbin 150069, People's Republic of China; State Key Laboratory of Veterinary Biotechnology, Harbin Veterinary Researched Institute, Chinese Academy of Agricultural Sciences, Harbin 150069, People's Republic of China; National High Containment Laboratory for Animal Diseases Control and Prevention, Harbin 150069, People's Republic of China

**Keywords:** SARS-CoV-2, mink, replication, pathogenicity, transmission, animal model

## Abstract

Minks are raised in many countries and have transmitted severe acute respiratory syndrome–coronavirus 2 (SARS-CoV-2) to humans. However, the biologic properties of SARS-CoV-2 in minks are largely unknown. Here, we investigated and found that SARS-CoV-2 replicates efficiently in both the upper and lower respiratory tracts, and transmits efficiently in minks via respiratory droplets; pulmonary lesions caused by SARS-CoV-2 in minks are similar to those seen in humans with COVID-19. We further found that a spike protein-based subunit vaccine largely prevented SARS-CoV-2 replication and lung damage caused by SARS-CoV-2 infection in minks. Our study indicates that minks are a useful animal model for evaluating the efficacy of drugs or vaccines against COVID-19 and that vaccination is a potential strategy to prevent minks from transmitting SARS-CoV-2.

**Introduction**

Coronaviruses (CoV) have caused three epidemics in the 21st century, including severe acute respiratory syndrome (SARS) in 2003 [1], Middle East respiratory syndrome (MERS) in 2012 [2], and the currently spreading COVID-19 [3, 4]. The causative pathogens for these three epidemics are SARS-CoV, MERS-CoV, and SARS-CoV-2, respectively. Unlike SARS-CoV and MERS-CoV, which each caused human infections over a limited geographic area, SARS-CoV-2 has caused a pandemic [5, 6, 7]. As of October 11, 2020, over 37 million human cases have been reported, and more than one million humans have died from the infection [8].

ORIGINAL UNEDITED MANUSCRIPT

The SARS-CoV-2 shares the highest homology at the nucleotide level with the coronavirus RaTG13, which was found in horseshoe bats (*Rhinolophus spp.*) in Yunnan province, China, in 2013 [9], implying that the SARS-CoV-2 and RaTG13 may have descended from a similar ancestor. Although it remains unknown which animal(s) served as intermediate hosts to transfer the virus from its original host to humans, it has been reported that multiple animal species, including ferrets, hamsters, nonhuman primates, cats, dogs, tigers, raccoon dogs, and minks are permissive for SARS-CoV-2 infection or have tested positive in the field [10, 11, 12, 13, 14, 15, 16, 17, 18, 19, 20, 21, 22, 23]. Among these susceptible animals, minks are the only species farmed on a large scale in many countries that have transmitted SARS-CoV-2 to humans, suggesting their potential to become a new reservoir of SARS-CoV-2 [20, 24]. However, the biologic properties of SARS-CoV-2 in minks are largely unknown. Here, we performed a detailed study to understand the replication, pathogenicity, and transmission of SARS-CoV-2 in minks. We also explored the potential of minks to serve as model animals for the development of COVID-19 control measures.

**Results**

All experiments with infectious SARS-CoV-2 were performed in the biosafety level 4 and animal biosafety level 4 facilities in the Harbin Veterinary Research Institute (HVRI) of the Chinese Academy of Agricultural Sciences (CAAS), which were approved for such use by the Ministry of Agriculture and Rural Affairs of China. Details of the biosafety and biosecurity measures taken were described previously [14]. The protocols for animal study and animal welfare were reviewed and approved by the Committee on the Ethics of Animal Experiments of the HVRI of CAAS (approval number 2020-05-01JiPi).

ORIGINAL UNEDITED MANUSCRIPT

**SARS-CoV-2 replicates efficiently in the upper and lower respiratory tracts of minks**

To investigate the replication and tissue tropism of SARS-CoV-2 in minks, three 13-month-old minks were inoculated intranasally (i.n.) with $5\times10^6$ plaque-forming units (PFU) in 1ml of the SARS-CoV-2 HRB25 strain, which was isolated from a patient in Harbin in February, 2020 [25]. Nasal washes, concha swabs, and rectal swabs were collected on days 2 and 4 post-inoculation (p.i.) for viral RNA and infectious virus detection. On day 4 p.i., the minks were euthanized and their nasal turbinates, soft palates, tonsils, tracheas, lungs, hearts, submaxillary lymph nodes, kidneys, spleens, livers, small intestines, and brains were collected for viral RNA and infectious virus detection.

Viral RNA was detected in the nasal washes of all three animals on days 2 and 4 p.i., and also from the ear swabs and rectal swabs of two animals on day 2 p.i. (Fig. 1A). Infectious virus was detected in the nasal washes of all three animals on days 2 and 4 p.i., but not from the concha swabs or rectal swabs of any animals at any time points (Fig. 1B). Viral RNA was detected in the nasal turbinates, soft palates, tonsils, all lung lobes, and submaxillary lymph nodes of all three minks, in the trachea of two minks, and in the ileum of one mink, but was not detected in any other organs of any animal tested (Fig. 1C). Infections virus was detected in most of the viral RNA-positive samples (Fig. 1D). These results demonstrate that SARS-CoV-2 can replicate efficiently in the upper and lower respiratory tracts of minks.

**SARS-CoV-2 causes severe pathological injury in the respiratory tracts of minks**

Nasal turbinate and lung samples of the minks that were euthanized on day 4 p.i. were also collected for pathological studies. Compared to the clean nasal cavity of the naïve control mink (fig.

ORIGINAL UNEDITED MANUSCRIPT

S1A) [26], the nasal cavities of the virus-inoculated minks were filled with mucinous-purulent secretion, which was composed of neutrophil debris and mucus (fig. S1, B and C). Inflammatory infiltrates, epithelial degeneration, and necrosis were observed in the nasal mucosa and submucosa of the vestibular region (fig. S1D), respiratory region (fig. S1E), and olfactory region (fig. S1F). In the nasal mucinous-purulent secretion of the infected animals, numerous positive signals indicating SARS-CoV-2 nucleoprotein antigen (fig. S1H) were noted. Positive signals were also detected in the epithelium of the nasal mucosa of the vestibular region, respiratory region, and olfactory region (fig. S1I, J, K) and that of the tracheal mucosa (fig. S1L). These data suggest a major role for nasal secretion in transmission and olfactory function impairment, as has been reported in SARS-CoV-2 infected human patients [27].

The lungs of the virus-infected minks showed severe lesions with extensive and diffuse consolidation (Fig. 2B). At higher magnification, various histopathological changes were observed, including fibrinous necrosis of the blood vessels (Fig. 2C), intra-alveolar serous and fibrin exudation (Fig. 2, D and E), hemorrhage (Fig. 2E), focal multinucleated syncytial cells (Fig. 2F), intra-alveolar and interalveolar septa proliferation and infiltration of macrophages (Fig. 2G), severe lymphoplasmacytic perivasculitis and vasculitis, and fibrin formation in blood vessels (Fig. 2, H and I). A few intranuclear inclusion bodies in monocyte-like cells (Fig. 2I), degeneration of bronchiolar epithelium (Fig. 2J), peribronchiolar inflammatory infiltrates (Fig. 2, J and K), and degeneration of bronchial gland epithelium (Fig. 2L) were also noted. Viral antigen-positive cells were detected in the bronchi and lungs of all animals on day 4 p.i. (Fig. 2, M, N, and O). There was marked proliferation of macrophages, monocytes, and neutrophils in the lungs, which was confirmed by

ORIGINAL UNEDITED MANUSCRIPT

immunohistochemical staining with an anti-MAC387 antibody (Fig. 2P). Robust deposition of intra- and inter-alveolar mature collagen fibers was detected (Fig. 2Q); mucous-fibrin mixture deposits were detected in the bronchial lumen (red staining, Fig. 2R) and in the blood vessels (fig. 2, S and T). Importantly, most of these pathologic changes have been observed in human COVID-19 patients [28, 29, 30], as summarized in table 1, suggesting that SARS-CoV-2 causes similar damage to the respiratory systems of minks and humans.

**SARS-CoV-2 easily transmits among minks via respiratory droplets**

To investigate the ability of SARS-CoV-2 to transmit among minks via respiratory drops, three animals were inoculated i.n. with $5\times10^{6}$ PFU of HRB25 and each animal was placed in a separate cage within an isolator. Twenty-four hours later, three naive minks were placed in each cage adjacent to the ones that held the virus-inoculated mink without direct contact. Nasal washes, and rectal and concha swabs were collected every other day from days 2 to 18 p.i. from the inoculated animals [and from days 1 to 17 post-exposure (p.e.) from the exposed animals] for viral RNA detection and virus titration. Body weights of all six animals were recorded every other day. In the three virus-inoculated minks, viral RNA was detected in the nasal washes of all three animals on days 2, 4, 6, and 8 p.i., and in the nasal washes of two animals and one animal on days 10 and 12 p.i., respectively, but not from any animals on days 14, 16, and 18 p.i. (Fig. 3A). Viral RNA was also detected in the concha swab of one mink on days 4 and 6 p.i. and in the rectal swab of another mink on day 8 p.i., (fig. S2A). Among the exposed minks, viral RNA was started to be detected in the nasal washes of one, two, and all three animals on days 3, 5 and 9 p.e., respectively (Fig. 3A). Viral RNA was also detected from the concha swab and rectal swab of one mink on day 11 p.i. and day 13 p.i., respectively (fig. S2, A and

ORIGINAL UNEDITED MANUSCRIPT

C). Infectious virus was detected in the nasal washes of the infected minks from days 2 to 8 p.i., and in the nasal washes of the exposed animals from days 5 to 11 p.e. (Fig. 3B), but was not detected in the concha swabs or rectal swabs of any of the minks (fig. S2, B and D). The inoculated minks experienced 10%–20% body weight loss during the 18-day observation period (Fig. 3C); one virus-inoculated mink experienced watery nasal discharge. The exposed minks lost about 5% of their body weight (Fig. 3D). All of the inoculated and exposed minks seroconverted on day 18 p.i. (Fig. 3, E and F). These data indicate that SARS-CoV-2 easily transmits among minks via respiratory droplets.

**A spike protein-based subunit vaccine protects minks against SARS-CoV-2**

The above studies indicate that minks are most similar to humans in terms of replication, transmission, and lung lesions after being infected with SARS-CoV-2, making minks a potential animal model for SARS-CoV-2 study and COVID-19 control measure evaluation. In addition, since minks are raised in many countries, actions are urgently needed to prevent farmed minks from becoming a reservoir of SARS-CoV-2. We therefore evaluated the protective efficacy of a spike protein-based subunit vaccine against SARS-CoV-2 in minks. Six 13-month-old minks were immunized intramuscularly with two doses of an aluminium-adjuvanted subunit vaccine; each dose contained 25 μg of the spike protein in a 0.5-ml volume, and there was a two-week interval between immunizations. Sera were collected at different time points from the vaccinated minks for detecting antibody against SARS-CoV-2. The animals were challenged intranasally with $5\times10^{6}$ PFU of HRB25 at five weeks post the second dose vaccine inoculation.

One and four of the six vaccinated minks developed antibodies to SARS-CoV-2 one week and

ORIGINAL UNEDITED MANUSCRIPT

two weeks after the first vaccination, respectively, and all of the six minks developed antibodies against SARS-CoV-2 after the second dose vaccination tested by an ELISA (fig. S3A). At five weeks after the second immunization, the neutralization antibody titers of the minks ranged from 64 to 256 (6log2 to 8log2) (fig. S3B). Three vaccinated minks and three control minks were euthanized on day 4 post-challenge (p.c.). Among the three vaccinated animals, viral RNA of the challenged virus was detected in the nasal turbinate, soft palate, and tonsil of one or two of the three animals, and in one lung lobe of one mink (Fig. 4A), but infectious virus was not detected in any organs tested (Fig. 4B). In contrast, high copy numbers of viral RNA and infectious virus were detected in all of the tested organs of the control minks, except for the tonsils, in which infectious virus was not detected (Fig. 4, A and B). Histological studies of lung samples from the vaccinated minks revealed mild perivasculitis with/without slight peribronchiolitis and interstitial pneumonitis (fig. S4, A to C), but viral antigen was not detected in the lungs of any of the minks (fig. S4, D to F). Lung samples from the control minks showed acute interstitial pneumonia, severe perivasculitis, and moderate peribronchiolitis (fig. S4, G to I); the lung parenchyma contained a large amount of diffuse viral antigen (fig. S4, J to L).

Nasal washes of the three remaining minks in each group were collected for viral RNA detection and infectious virus titration. Viral RNA was detected in the nasal washes of the control minks on days 2, 4, 6, 8, 10, and 12 p.c., but was only detected in the nasal washes of the vaccinated minks on days 2, 4, and 6 p.c. with significantly low levels (Fig. 4C). Infectious virus was detected in the nasal washes of the control minks on days 2, 4, 6, and 8 p.c., but was only detected in the nasal wash of one vaccinated mink on day 2 p.c. (Fig. 4D).These studies show that the spike protein-based subunit

ORIGINAL UNEDITED MANUSCRIPT

vaccine largely prevented the replication of SARS-CoV-2 in the upper and lower respiratory tracts of the minks, and prevented SARS-CoV-2-induced lung damage in minks.

**Discussion**

In summary, we performed detailed studies to investigate the replication, transmission, and pathogenicity of SARS-CoV-2 in minks and found that SARS-CoV-2 replicates efficiently in both the upper and lower respiratory tracts and causes severe lesions in the nasal mucosa and lungs of minks. In addition, we found that SARS-CoV-2 transmits efficiently in minks via respiratory droplets. Oreshkova *et al*. reported that SARS-CoV-2 was lethal in farmed pregnant minks [20]; however, in the present study, we found that virus-inoculated minks lost 10%–20% of their body weight at around day 8 p.i., and exposed minks lost about 5% of their body weight, but none of the virus-inoculated or -exposed minks died. The exposed animals lost less body weight than the virus-inoculated animals, largely because the initial dose received by the exposed animals was much lower than that received by the inoculated animals, although the titer of the virus in the exposed animals reached a peak level similar to that of the inoculated animals. These findings suggest that pregnant minks may be more vulnerable to SARS-CoV-2 infection than non-pregnant ones. Given that minks are highly susceptible to SARS-CoV-2 and could be easily infected by this widely spreading virus, surveillance and active control measures in minks should be implemented to prevent minks from becoming reservoirs of SARS-CoV-2.

Animal models are needed for the evaluation of vaccines and antiviral drugs for COVID-19. Human angiotensin-converting enzyme 2 (hACE-2) receptor transgenic mice have been generated and proved to be susceptible to SARS-CoV-2 infection, resulting in a range of clinical signs with mild to fatal disease depending on the transgene [31, 32, 33, 34]. SARS-CoV-2 adaptation to mice has also

ORIGINAL UNEDITED MANUSCRIPT

been attempted through either serial passaging or reverse genetics [35, 36, 37]. Non-human primates, including rhesus macaques, African green monkeys, and marmosets, have been shown to be susceptible to SARS-CoV-2 infection [38, 39, 40, 41]. Ferrets have also been shown to be susceptible to SARS-CoV-2 infection resulting in mild disease with shedding from the upper respiratory tract [14, 17, 42, 43].

Both minks and ferrets are mustelid and are highly susceptible to SARS-CoV-2; however, the replication patterns of SARS-CoV-2 in ferrets and minks are different. Studies shown that the host factors, such as ACE2 [9, 44, 45, 46] and neuropilin-1 [47, 48], play important roles in the SARS-CoV-2 replication, it remains to be investigated if ferrets and minks have different expression levels of these critical host factors in their organs. SARS-CoV-2 efficiently replicates in the upper respiratory tract of ferrets, but its replication in the lungs of ferrets is undetectable [14]. Therefore, the ferret model can only be used to assess the efficacy of vaccines or antivirals in preventing or reducing virus replication in the upper respiratory tract; it cannot be used to evaluate whether they reduce or eliminate lung lesions. SARS-CoV-2 replicates in the upper and lower respiratory tracts of minks and causes lung lesions highly similar to those seen in human COVID-19 patients [28, 30]. Accordingly, using minks as model animals will enable us to more precisely evaluate the efficacy of antiviral drugs or vaccines for COVID-19.

Minks are raised on a large scale in many countries and are at high risk of SARS-CoV-2 infection. In fact, SARS-CoV-2 infection of minks has been reported in several countries in Europe and the Americas, and millions of minks have been culled to control the spread of the virus [24]. In the present study, we found that a spike protein-based subunit vaccine largely prevented SARS-CoV-2 replication and protected minks from SARS-CoV-2-induced lung damage. Our previous studies with H7N9

ORIGINAL UNEDITED MANUSCRIPT

influenza viruses have showed that vaccinated animals could be perfectly protected from exposure to virus infected animals, even though the vaccine cannot provide complete protection when the animals were challenged by direct infection [49, 50]. Given that the vaccine provided sound protection to high dose of SARS-CoV-2 challenge, it is reasonable to speculate that the vaccinated animals would be protected from infection of natural exposure. Vaccination may, therefore, be an option for preventing SARS-CoV-2 infection of minks.

## Materials and Methods

The detailed descriptions of materials and methods are available as Supporting information at NSR online.

## Data and materials availability

All data are available in the manuscript or the supplementary materials.

## Acknowledgments

We thank S. Watson for editing the manuscript.

## Funding

This work was supported by the National Key R&D Program of China (2018YFC1200601, 2020YFC0846500, 2020YFC0841100) and by the Applied Technology Research and Development Project of Heilongjiang Province, China (GA20C006).

**Author contributions:** L.S., G.Z., Q.Y., Z.W., C.W, X. H., R. L., J.W., Q.Z., Y.L., N.H., J.D., J.B., H.W., and Y.G. performed the experiments; K.T., N.X., H.C., Z.B. designed the study and analyzed the data; H.C. and Z.B. wrote the manuscript.

**Conflict of interest statement.** None declared.

ORIGINAL UNEDITED MANUSCRIPT

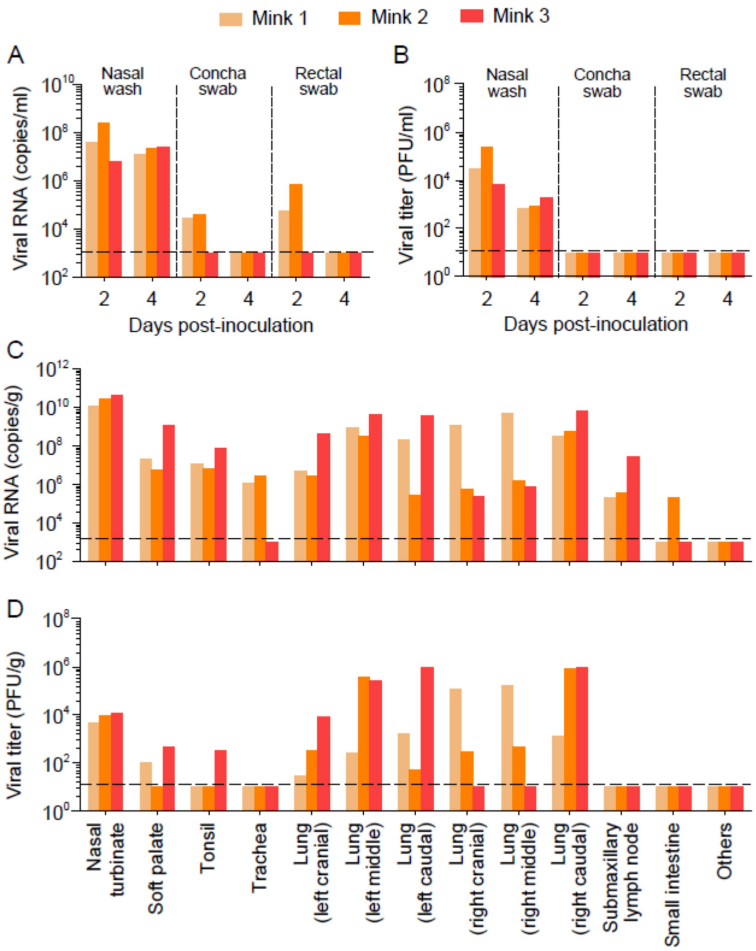

**Figure 1. Replication of SARS-CoV-2 in minks.** Three minks were inoculated with HRB25 virus, and their nasal washes, concha swabs, and rectal swabs were collected on days 2 and 4 post-infection (p.i.); organs were collected on day 4 p.i. for viral RNA detection and virus titration. (**A**) Viral RNA and (**B**) viral titers of nasal washes, concha swabs, and rectal swabs of minks on days 2 and 4 p.i. (**C**) Viral RNA and (**D**) viral titers of organs of minks on day 4 p.i. "Others" represents viral-negative organs, including the heart, kidneys, spleen, liver, pancreas and brain. Each color bar represents the value from an individual animal. The dashed lines indicate the lower limit of detection.

ORIGINAL UNEDITED MANUSCRIPT

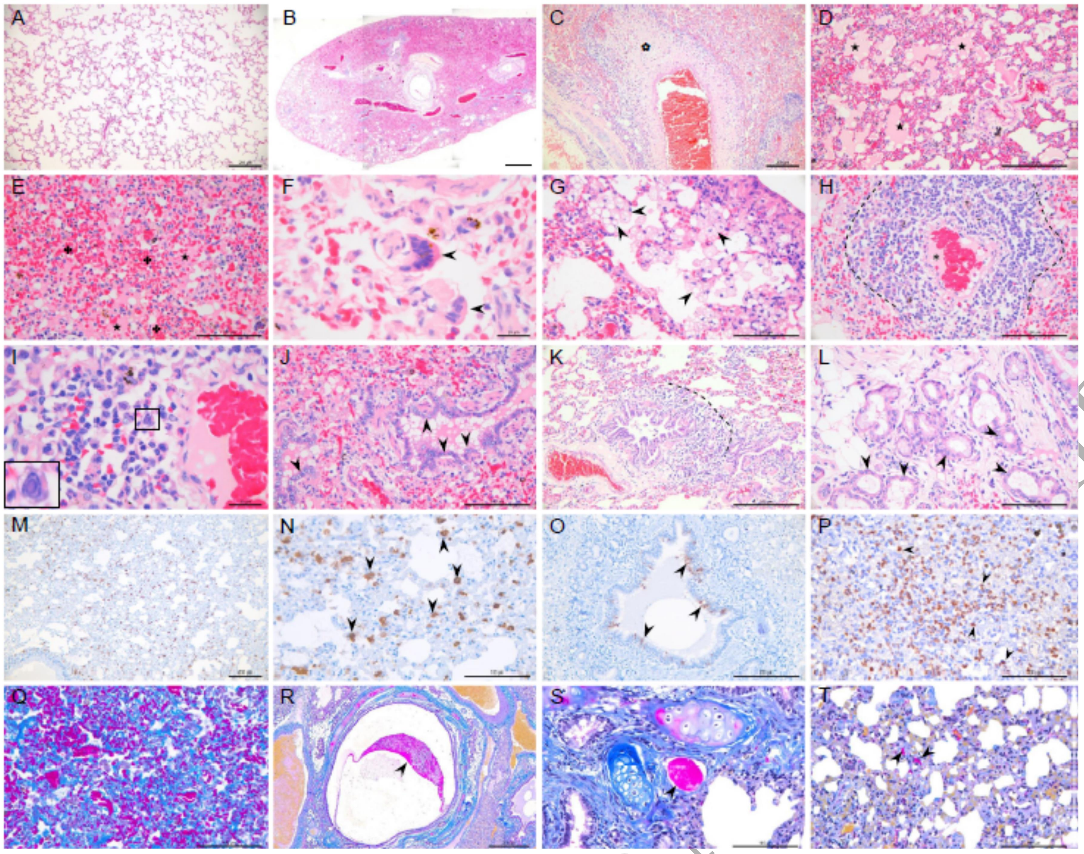

**Figure2. Histopathologic, immunohistochemical, and histochemical studies.** (**A**) Lung from control mink shows normal structure. (**B-T**) Lungs of minks infected with SARS-CoV-2. (**B**) Extensive and diffuse consolidation at sub-gross level. (**C**) Fibrinous necrosis of blood vessel wall (cinquefoil). (**D**) Intra-alveolar serous and fibrin exudation (asterisk). (**E**) Intra- and inter-alveolar hemorrhages (cloverleaf) and serous-fibrin exudation (asterisk). (**F**) Multinucleated syncytial cell in alveolar space (arrow). (**G**) Intra- and inter-alveolar foamy macrophage proliferation (arrow). (**H**) Severe lymphoplasmacytic perivasculitis and vasculitis (dotted line), and fibrin formation (asterisk) in a blood vessel. (I) Eosinophilic intranuclear inclusion body formation in a monocyte-like cell (□). (**J**) Degeneration of bronchiolar epithelium (arrow). (**K**) Peribronchiolar inflammatory infiltrate (dotted line). (**L**) Degeneration of bronchial gland epithelium. (**M, N**) Viral antigen-positive cells in the lung (arrow) and in (**O**) bronchi epithelium (arrow). (**P**) Marked proliferation of macrophages, monocytes, and neutrophils, confirmed by immunohistochemistry with an anti-MAC387 antibody (arrow). (**Q**) Deposition of intra- and inter-alveolar collagen confirmed by Masson trichrome (blue) staining. (**R**) Embolus of mucous-fibrin mixture in bronchial lumen confirmed by MSB staining (Red arrow). (**S**)

ORIGINAL UNEDITED MANUSCRIPT

Fibrin formation in a blood vessel confirmed by MSB staining (Red arrow). (**T**) Formation of microvascular thrombi confirmed by MSB staining (Red arrow). Scale bar in A, C, D, K, M, and O = 200 μm; in E, G, H, J, L, N, P, Q, S, and T = 100 μm; in F and I = 20 μm; and in R = 500 μm.

ORIGINAL UNEDITED MANUSCRIPT

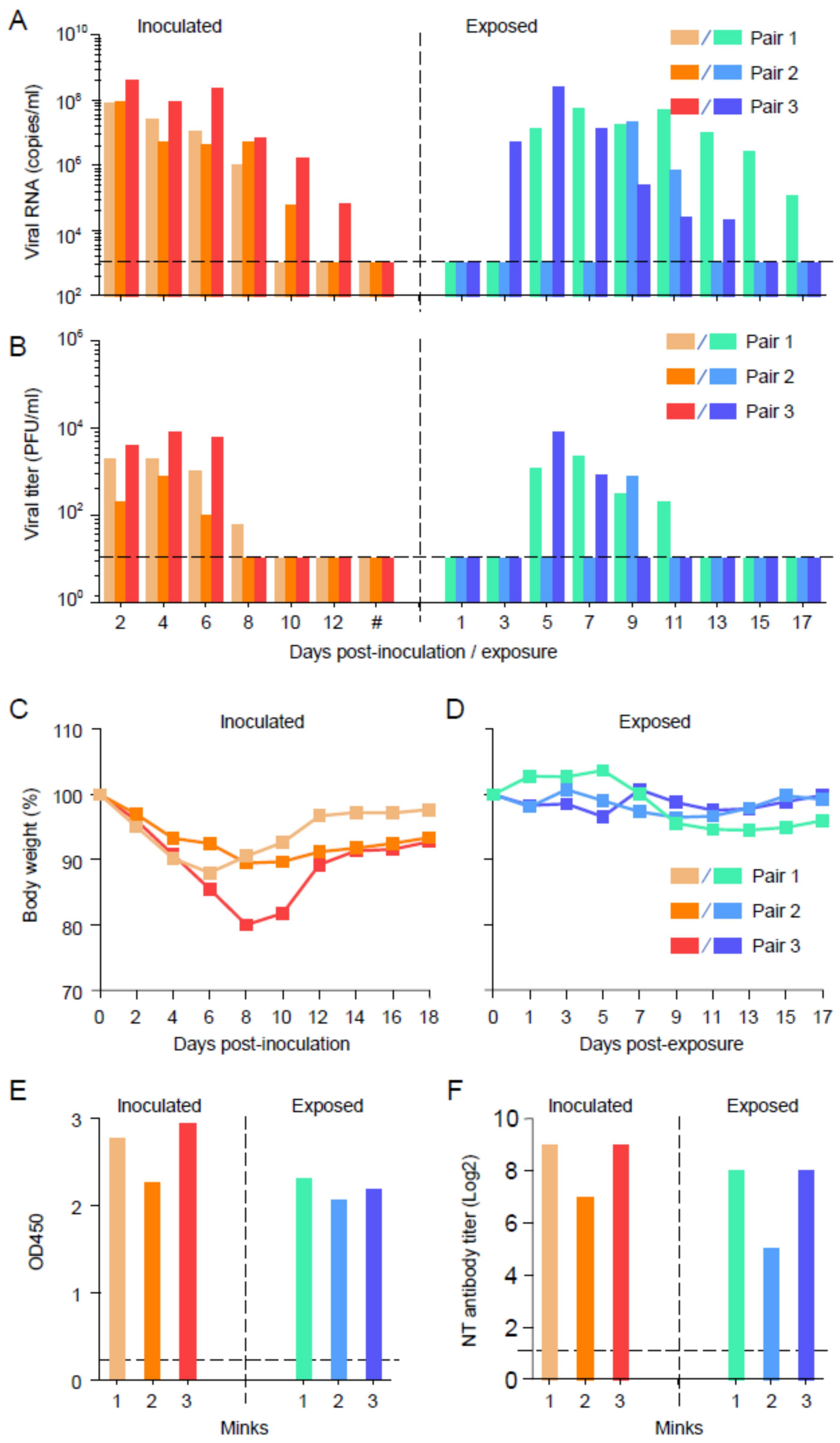

**Figure 3. Transmission of SARS-CoV-2 in minks.** Respiratory droplet transmission of SARS-CoV-2 HRB25 was evaluated in three pairs of minks. (**A**) Viral RNA in nasal washes of minks. (**B**) Viral titers in nasal washes of minks. (**C**) Body weight change of inoculated minks. (**D**) Body weight change of

ORIGINAL UNEDITED MANUSCRIPT

exposed minks. Antibodies against SARS-CoV-2 were detected by ELISA (**E**) and a neutralization assay (**F**). Hash represents the time points of days 14, 16, and 18 post-inoculation. Each color bar represents the value from an individual animal. The horizontal dashed lines indicate the lower limit of detection. OD450, optical density measured at 450 nm; NT antibody, neutralizing antibody.

ORIGINAL UNEDITED MANUSCRIPT

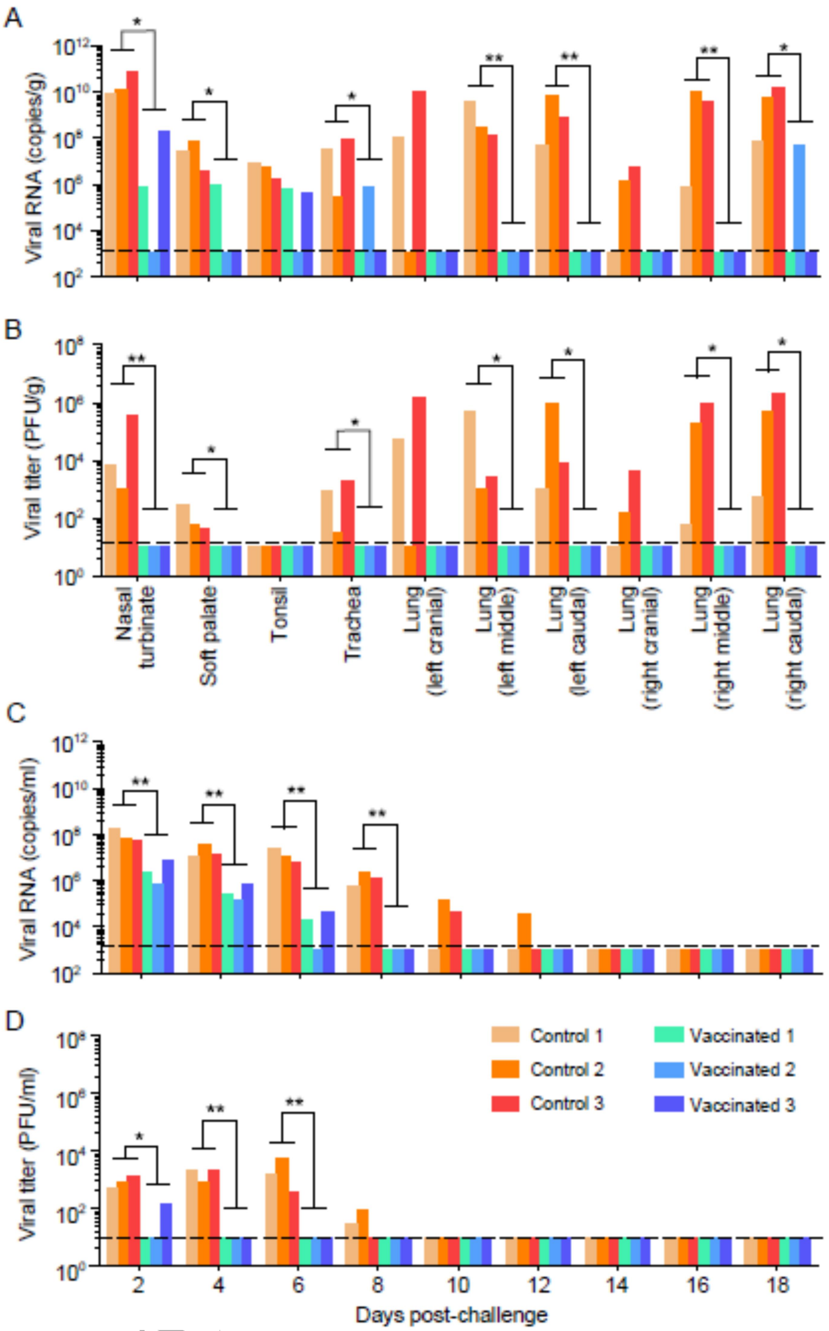

**Figure 4. Protective efficacy of a spike-based subunit vaccine against SARS-CoV-2 challenge in minks.** Six vaccinated or control minks were challenged with SARS-CoV-2 HRB25. Three minks from each group were euthanized on day 4 post-challenge (p.c.) and their organs were collected for viral RNA detection (**A**) and infectious virus titration (**B**); nasal washes of the three other remaining minks in each group were collected for viral RNA detection (**C**) and infectious virus titration (**D**). The

ORIGINAL UNEDITED MANUSCRIPT

horizontal dashed lines indicate the lower limit of detection. Viral RNA and viral titers of control and vaccinated minks were statistically analyzed by using the one-tailed unpaired t-test. Single asterisk indicates $P < 0.05$, and double asterisks indicates $P < 0.01$.

ORIGINAL UNEDITED MANUSCRIPT

**Table 1. Pathological characteristics observed in the lungs of minks infected with SARS-CoV-2 HRB25 strain.**

| Lesions observed in minks | | Percentage of 129 COVID-19 human patients having the lesions on autopsy as reported by Polak et al. [27] |
|---|---|---|
| Epithelial | Diffuse alveolar damage | 75% |
| | Desquamation and/or reactive hyperplasia of pneumocytes | 72% |
| | Multinucleated giant cells | 20% |
| | Viral inclusion bodies | 20% |
| Vascular | Capillary congestion | 45% |
| | (Micro) thrombi | 39% |
| | Alveolar hemorrhage | 33% |
| | Intra-alveolar fibrinous exudates | 26% |
| | Peri- or intravascular inflammatory infiltrates | 9% |
| | Interstitial fibrous changes, septal collagen deposition | 33% |
| Other | Interstitial and intra-alveolar inflammatory infiltrates | 64% |
| | Intra-alveolar edema | 46% |

ORIGINAL UNEDITED MANUSCRIPT
