# Supplementary material for: Replication, pathogenicity, and transmission of SARS-CoV-2 in minks
Source: Natl Sci Rev. 2020 Dec 8;8(3):nwaa291. doi: 10.1093/nsr/nwaa291 (PMC7798852; doi:10.1093/nsr/nwaa291)
Supplement: nwaa291_Supplemental_File [file nwaa291_supplemental_file.zip › Shuai_Table_1_R1.pdf]

Table 1. Pathological characteristics observed in the lungs of minks infected with SARS-CoV-2 HRB25 strain.

Lesions observed in minks		Percentage of 129 COVID-19 human patients having the lesions on autopsy as reported by Polak et al. [27]
Epithelial	Diffuse alveolar damage	75%
	Desquamation and/or reactive hyperplasia of pneumocytes	72%
	Multinucleated giant cells	20%
	Viral inclusion bodies	20%
Vascular	Capillary congestion	45%
	(Micro) thrombi	39%
	Alveolar hemorrhage	33%
	Intra-alveolar fibrinous exudates	26%
	Peri- or intravascular inflammatory infiltrates	9%
	Interstitial fibrous changes, septal collagen deposition	33%
Other	Interstitial and intra-alveolar inflammatory infiltrates	64%
	Intra-alveolar edema	46%
