# Supplementary material for: Replication, pathogenicity, and transmission of SARS-CoV-2 in minks
Source: Natl Sci Rev. 2020 Dec 8;8(3):nwaa291. doi: 10.1093/nsr/nwaa291 (PMC7798852; doi:10.1093/nsr/nwaa291)
Supplement: nwaa291_Supplemental_File [file nwaa291_supplemental_file.zip › Shuai_Supplementary_Materials.docx]

**SUPPLEMENTARY MATERIALS**

**Abbreviations**

**Materials and Methods**

**Figs. S1 to S4**

**Abbreviations**

CHO, Chinese Hamster Ovarian; CoV, Coronaviruses; COVID-19, Coronavirus Disease 2019; H&E, hematoxylin-eosin; HRB25, SARS-CoV-2/HRB25/human/2020/CHN (GISAID access no. EPI_ISL_467430); i.n., intranasally; MERS, Middle East respiratory syndrome; MSB, Martius scarlet blue; OD, optical density; p.c., post-challenge; p.e., post-exposure; p.i., post-inoculation; PFU, plaque-forming units; SARS, Severe acute respiratory syndrome; SARS-CoV-2, Severe acute respiratory syndrome–coronavirus 2.

**Materials and Methods**

**Facility, Ethics, and Biosafety statement**

All experiments with infectious SARS-CoV-2 were performed in the biosafety level 4 and animal biosafety level 4 facilities in the Harbin Veterinary Research Institute (HVRI) of the Chinese Academy of Agricultural Sciences (CAAS), which is approved for such use by the Ministry of Agriculture and Rural Affairs of China. The animal studies were carried out in strict accordance with the recommendations in the Guide for the Care and Use of Laboratory Animals of the Ministry of Science and Technology of the People’s Republic of China. The protocols were approved by the Committee on the Ethics of Animal Experiments of the HVRI of CAAS (approval number 2020-05-01JiPi).

**Cells and viruses**

Vero E6 cells were maintained in Dulbecco’s modified Eagle’s medium (DMEM) containing 10% fetal bovine serum (FBS) and antibiotics and incubated at 37 °C with 5% CO_2_. SARS-CoV-2/HRB25/human/2020/CHN (HRB25, GISAID access no. EPI_ISL_467430) was isolated from a patient in Vero E6 cells as reported previously [25]. Viral stocks were prepared in Vero E6 cells with DMEM containing 5% FBS. Infectious virus titers were determined by using a plaque forming unit (PFU) assay in Vero E6 cells.

**Vaccine preparation**

The full-length ectodomain (amino acids 1–1207, MN908947.3, with the furin cleavage site deleted) of the SARS-CoV-2 spike protein (S-protein) with the trimerization domain of T4 fibritin fused at the C-terminus [31] was expressed in Chinese Hamster Ovarian (CHO) cells. The S-protein trimer was purified by using a Ni Sepharose-HP column and concentrated by using the BCA method. A modified version of an aluminum hydroxyphosphate adjuvant [32, 33] was used in this study. The spike protein, in an isotonic solution with 100 mM sodium acetate buffer (pH 6.0), was mixed gently but thoroughly with the aluminum hydroxyphosphate adjuvant, allowed to settle, and then stored at 2–8 °C for at least one day prior to injection into animals. The final spike protein concentration in the vaccine was 50 μg/ml.

**Mink studies**

Thirteen-month-old male/female mixed outbred minks from local farms were used in this study. Randomization and blinding were not used for the allocation of animals to experimental groups. The number of animals used in each study was similar to that reported in previous publications [18, 24, 34] or was determined by following the “minimum-quantity-principle” in our protocol. All of the animal studies were performed once, and the biological replicates (i.e., number of animals used in each experiment) for each study is indicated below.

To evaluate the replication of SARS-CoV-2, three minks were intranasally (i.n.) inoculated with 5×10^6^ PFU of HRB25. On day 4 post-inoculation (p.i.), animals were euthanized and their organs, including nasal turbinates, soft palates, tonsils, tracheas, lungs, hearts, submaxillary lymph nodes, kidneys, spleens, livers, small intestines, and brains, were collected for viral RNA detection by qPCR, virus titration in Vero E6 cells, and histological studies.

To investigate the transmissibility of SARS-CoV-2 in minks, three animals were inoculated i.n. with 5×10^6^ PFU of HRB25 and each animal was placed in a separate cage within an isolator. Twenty-four hours later, three similar-aged naive minks were placed in each cage adjacent to the ones that held the virus-inoculated minks. Nasal washes, and rectal and concha swabs were collected and body weights were monitored every other day from days 2 to 18 p.i. (inoculated animals) or from days 1 to 17 post-exposure (p.e.) (exposed animals) for viral RNA detection and virus titration. Clinical signs were observed daily. Sera were collected on day 18 p.i. and antibodies against SARS-CoV-2 were detected by using the Double Antigen Sandwich ELISA kit (Luoyang Putai Biotechnology, Luoyang, China) and a neutralization assay.

To evaluate the protective efficacy of the spike-based subunit vaccine in minks, six minks were immunized intramuscularly with two doses of an aluminium-adjuvanted subunit vaccine. Each dose contained 25 μg of the spike protein in a 0.5-ml volume, administered with a two-week interval between doses. Six similar-aged naive minks were used as controls. Five weeks after the second inoculation, sera from the vaccinated animals were collected for antibody detection, and all of the minks were then challenged i.n. with 5×10^6^ PFU of HRB25. Three animals from each group were euthanized on day 4 p.c., and their organs were collected for viral RNA detection and virus titration. Nasal washes were collected every other day from the other three minks in each group to monitor virus shedding for 18 days. Viral RNA and viral titers of control and vaccinated minks were statistically analyzed by using the one-tailed unpaired t-test. *P* values of < 0.05 were considered significant.

**qPCR**

Viral genomic RNA of SARS-CoV-2 was extracted by using a QIAamp vRNA Mini kit (Qiagen, Hilden, Germany). Reverse transcription was performed by using the HiScript II Q RT SuperMix (Vazyme, Nanjing, China) for qPCR. qPCR was performed to quantitate the number of viral N gene RNA copies by using the Applied Biosystems QuantStudio 5 Real-Time PCR System (Thermo Fisher Scientific, Waltham, MA, USA) with Premix Ex Taq (probe qPCR) (Takara, Dalian, China). The *N* gene-specific primers (forward, 5'- GGGGAACTTCTCCTGCTAGAAT-3'; reverse, 5'-CAGACATTTTGCTCTCAAGCTG-3') and probe (5'-FAM-TTGCTGCTGCTTGACAGATT-TAMRA-3') were utilized according to the information provided by the National Institute for Viral Disease Control and Prevention, China (http://nmdc.cn/nCoV). The amount of vRNA for the target SARS-CoV-2 *N* gene was normalized to a standard curve obtained by using a plasmid (pBluescriptIISK-N, 4,221 bp) containing the full-length cDNA of the SARS-CoV-2 *N* gene.

**ELISA**

Antibodies against SARS-CoV-2 were detected by using a Double Antigen Sandwich ELISA Kit (Luoyang Putai Biotechnology, Luoyang, China) according to the manufacturer’s instructions. Briefly, 100 μl of sera were added to antigen-precoated microtiter plates and incubated at 37 °C for 30 min. Plates were then washed 5 times with wash buffer, and incubated with HRP-conjugated antigen at 37 °C for 30 min. Plates were washed 5 times with PBST, and 100 μl of substrate solution (50 μl of Chromogen solution A and 50 μl of Chromogen solution B) was added to enable colorimetric analysis. The reaction was stopped by adding 50 μl of stop buffer, and optical density (OD) was measured at 450 nm.

**Plaque reduction neutralization test**

SARS-CoV-2 HRB25 strain (100 PFU) was incubated with two-fold serial dilutions of sera for 1 h at 37 °C. A plaque assay was then performed in Vero E6 cells with the neutralization mixtures. Neutralizing antibody titers were calculated as the maximum serum dilution yielding a 50% reduction in the number of plaques relative to control serum prepared from uninfected minks.

**Histopathologic and immunohistochemical studies**

Immediately after the nasal turbinates and lungs were isolated, they were fixed in 10% neutral-buffered formalin. After the tissues were adequately fixed, the nasal turbinates were decalcified with 10% formic acid. The tissues were then processed, that is, subjected to dehydration, xylene penetration, paraffin infiltration, and paraffin embedding. Sections (4-μm thick) were stained with hematoxylin-eosin (H&E) for routine histologic examination. Sections were also stained with special stains, including Masson’s trichrome (Baso Diagnostics Inc., Zhuhai, Guangdong, China) for collagen, and Martius scarlet blue (MSB, Solarbio, Beijing, China) for fibrin.

The sections used for immunohistochemistry were dewaxed in xylene and hydrated through a series of descending concentrations of alcohol to water. For viral antigen retrieval, sections were immersed in citric acid/sodium citrate solution at 121°C for 15 minutes. After cooling, the sections were treated with 3% hydrogen peroxide for 30 minutes to remove endogenous peroxidase activity and were blocked with 8% skim milk to reduce nonspecific binding. After three 5-minute washes in TBS, the sections were incubated with a rabbit anti-SARS-CoV-2 nucleoprotein monoclonal antibody (1:500; Frdbio. Wuhan, China; catalog number: nCov-N-rmAb) or mouse anti-S100A9 + calprotectin monoclonal antibody (clone MAC387, code ab22506, Abcam, Cambridge, UK) in 8% skim milk at 4°C overnight. The sections were then washed again with TBS and incubated with an anti-rabbit IgG (whole molecule)-HRP (1:600, Sigma-Aldrich, catalog number: A-9169) or an anti‐mouse IgG (H+L) secondary antibody-HRP (1:400; Invitrogen, catalog number: 31430) at room temperature for 60 minutes. Immunostaining was visualized with DAB and counterstained with hematoxylin.

**Supplementary figure legends**

**Supplementary Figure S1. Histopathologic and immunohistochemical studies on nasal turbinate and trachea of minks that were euthanized on day 4 post-SARS-CoV-2 infection.** (**A**) Nasal turbinate of control mink showing clean nasal passage (asterisk). In the virus-infected minks, mucous-purulent secretion and cellular debris in nasal passage (asterisk) (**B**) and necrosis of epithelial cells (arrow head) (**C**) were observed. Inflammatory infiltrate and epithelial damage (asterisk) in the nasal vestibular region (**D**), severe inflammatory infiltrate (asterisk) and epithelial damage (arrowhead) in the nasal respiratory region (**E**), inflammatory infiltrate (asterisk), and epithelial damage (arrowhead) in the nasal olfactory region (**F**) were observed. No positive viral N protein signals were detected in the nasal mucosa of the control mink (**G**), where as many positive viral N protein signals were detected in the secretion in the nasal cavity (arrow) (**H**), in the epithelium of the nasal vestibular region (arrow) (**I**), in the epithelium of the nasal respiratory region (arrow) (**J**), and in the epithelium of the nasal olfactory region (arrow) of the virus-infected minks (**K**). A few positive viral N protein signals were also detected in the tracheal epithelium (arrow) of the virus-infected minks (L). A to F, stained with H&E, G to L, stained with an anti-SARS-CoV-2 nucleoprotein monoclonal antibody. Scale bar in A and G = 1000 μm; in B, H, and I = 200 μm; and in C–F, J–L = 100 μm.

**Supplementary Figure S2. Viral RNA and infectious virus detection in the concha swabs and rectal swabs of minks in the transmission study.** Respiratory droplet transmission of SARS-CoV-2 HRB25 was evaluated in three pairs of minks. (**A**) Viral RNA in concha swabs of minks. (**B**) Viral titers in rectal swabs of minks. (**C**) Viral RNA in concha swabs of minks. (**D**) Viral titers in rectal swabs of minks. Hash represents days 14, 16, and 18 post-inoculation. Each color bar represents the value from an individual animal. The horizontal dashed lines indicate the lower limit of detection.

**Supplementary Figure S3. Antibody titers in minks that were vaccinated with two doses of 25 μg of spike protein-based subunit vaccine.** Sera were collected from minks at the indicated timepoints after vaccination and antibodies against SARS-CoV-2 were detected by ELISA (**A**), the sera that were collected at five weeks post the second immunization were also tested by a neutralization assay (**B**). Each color bar represents the value from an individual animal. The horizontal dashed lines show the cutoff value for seroconversion. OD450, optical density measured at 450 nm; NT antibody, neutralizing antibody.

**Supplementary Figure S4. Histopathologic and immunohistochemical studies on lungs of vaccinated and control minks that were euthanized on day 4 post-SARS-CoV-2 challenge.** Mild perivasculitis with/without slight peribronchiolitis and interstitial pneumonitis were observed in the lungs of the three vaccinated minks (**A-C**), and viral antigen was not detected in the lungs of any minks (**D–F**). In the three control minks, acute interstitial pneumonia with severe perivasculitis and moderate peribronchiolitis were observed (**G–I**), and massive amounts of viral antigen were diffusively located throughout the lungs (**J–L**). A–C and G–I, stained with H&E**.** D–F and J–L, stained with an anti-SARS-CoV-2 nucleoprotein monoclonal antibody. Scale bar = 200 μm.
