# Supplementary material for: Replication, pathogenicity, and transmission of SARS-CoV-2 in minks
Source: Natl Sci Rev. 2020 Dec 8;8(3):nwaa291. doi: 10.1093/nsr/nwaa291 (PMC7798852; doi:10.1093/nsr/nwaa291)
Supplement: nwaa291_Supplemental_File [file nwaa291_supplemental_file.zip › Shuai_Table_1_R1.docx]

**Table 1. Pathological characteristics observed in the lungs of minks infected with SARS-CoV-2 HRB25 strain.**

| Lesions observed in minks | | Percentage of 129 COVID-19 human patients having the lesions on autopsy as reported by Polak et al. [27] |
| --- | --- | --- |
| Epithelial | Diffuse alveolar damage | 75% |
|  | Desquamation and/or reactive hyperplasia of pneumocytes | 72% |
|  | Multinucleated giant cells | 20% |
|  | Viral inclusion bodies | 20% |
| Vascular | Capillary congestion | 45% |
|  | (Micro) thrombi | 39% |
|  | Alveolar hemorrhage | 33% |
|  | Intra-alveolar fibrinous exudates | 26% |
|  | Peri- or intravascular inflammatory infiltrates | 9% |
|  | Interstitial fibrous changes, septal collagen deposition | 33% |
| Other | Interstitial and intra-alveolar inflammatory infiltrates | 64% |
|  | Intra-alveolar edema | 46% |
