# Supplementary figures and images for: Replication, pathogenicity, and transmission of SARS-CoV-2 in minks
Source: Natl Sci Rev. 2020 Dec 8;8(3):nwaa291. doi: 10.1093/nsr/nwaa291 (PMC7798852; doi:10.1093/nsr/nwaa291)

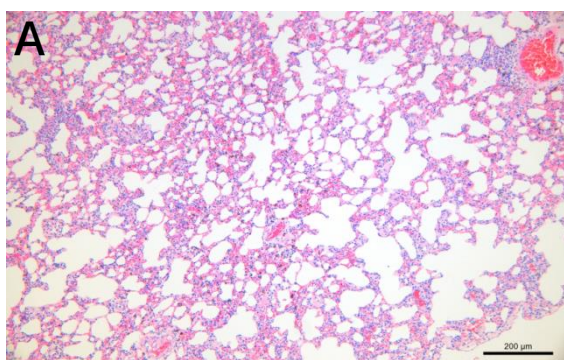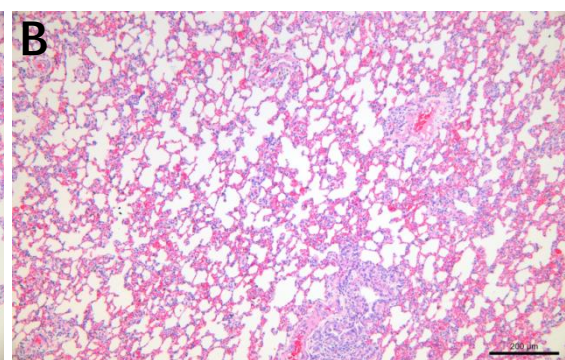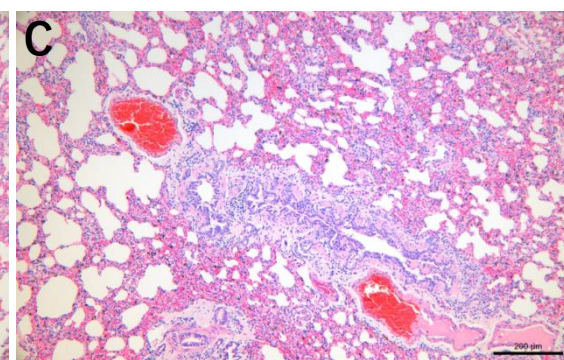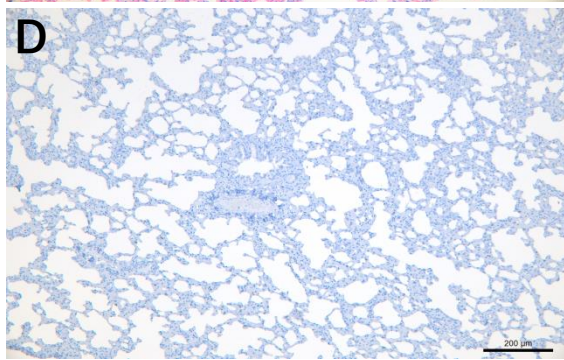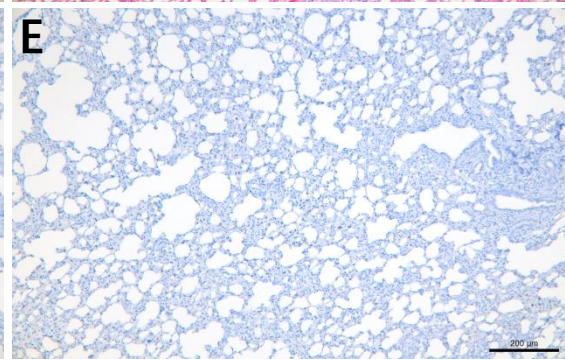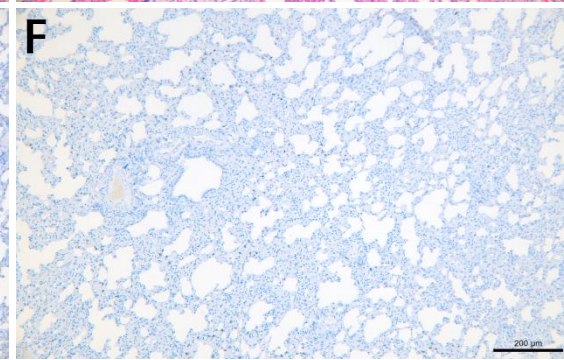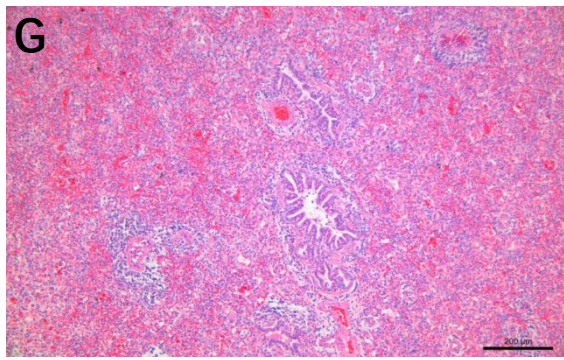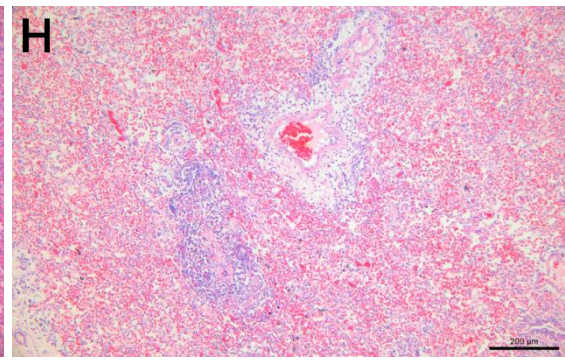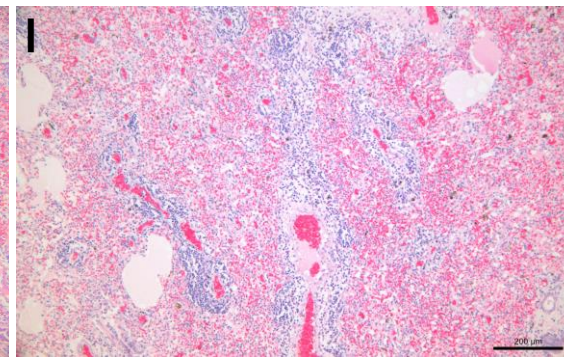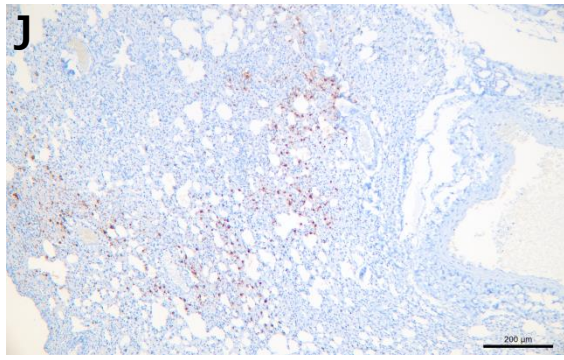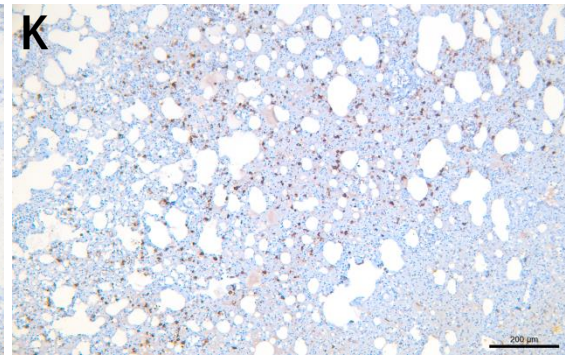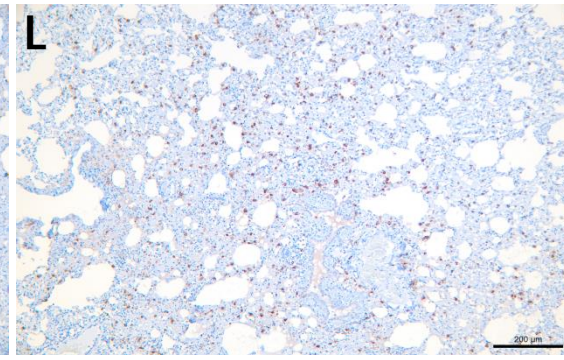

Supplement: nwaa291_Supplemental_File [file nwaa291_supplemental_file.zip › Fig_S4.pdf]

Figure  
S1

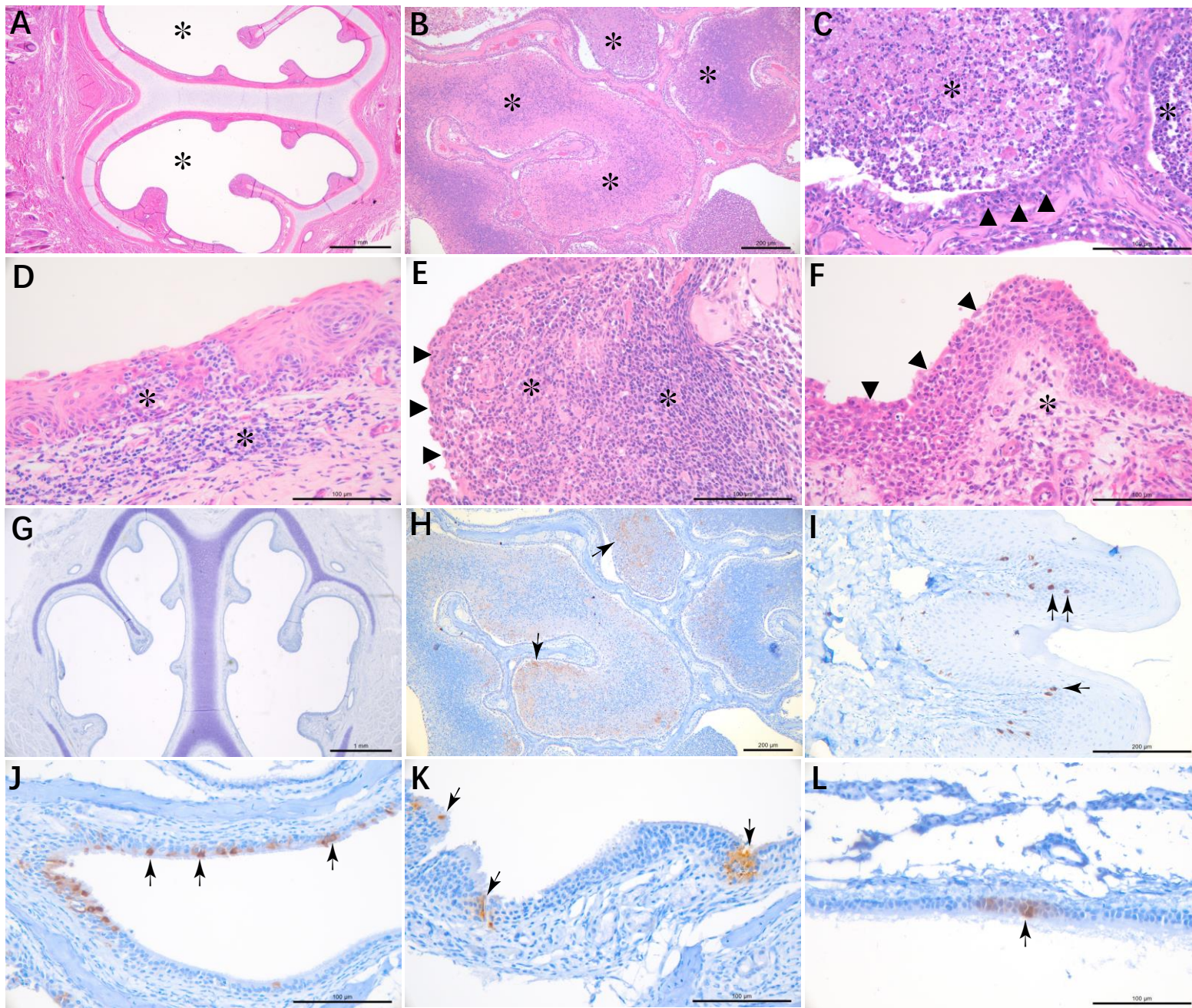

Supplement: nwaa291_Supplemental_File [file nwaa291_supplemental_file.zip › Figure_S1.pdf]

## Concha swabs

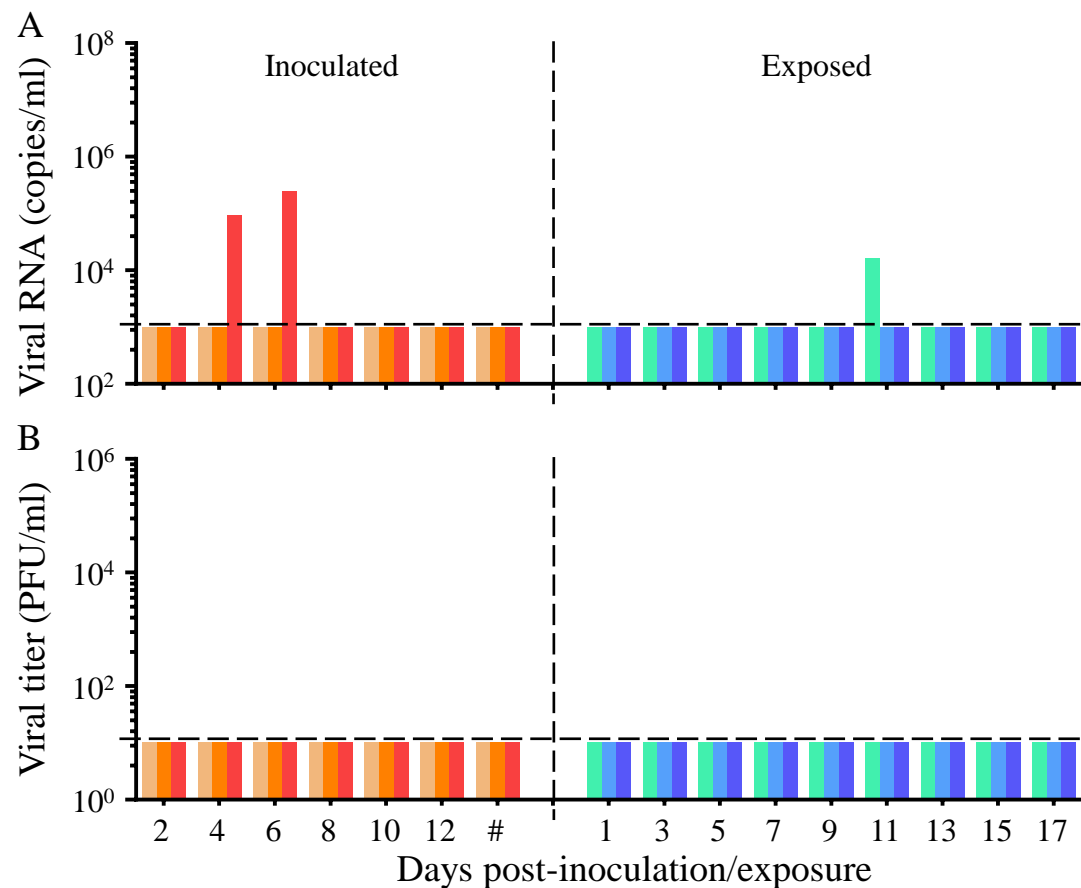

## Rectal swabs

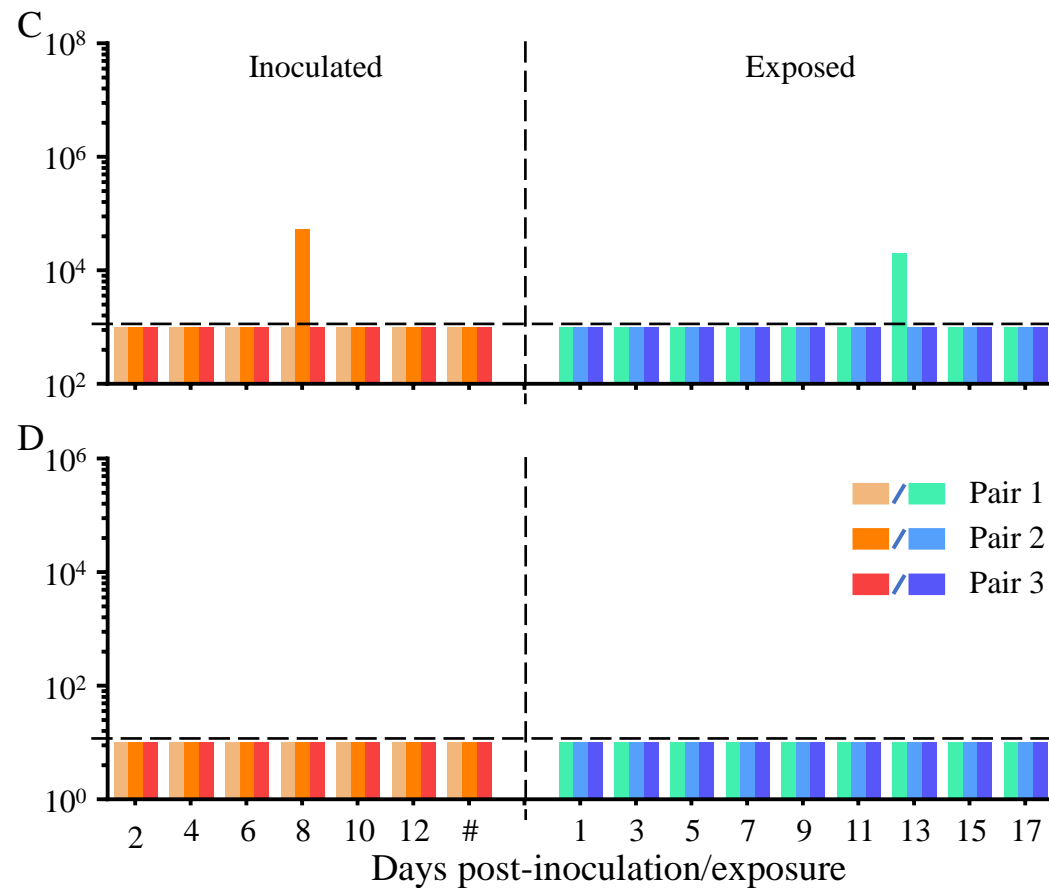

Supplement: nwaa291_Supplemental_File [file nwaa291_supplemental_file.zip › Figure_S2.pdf]

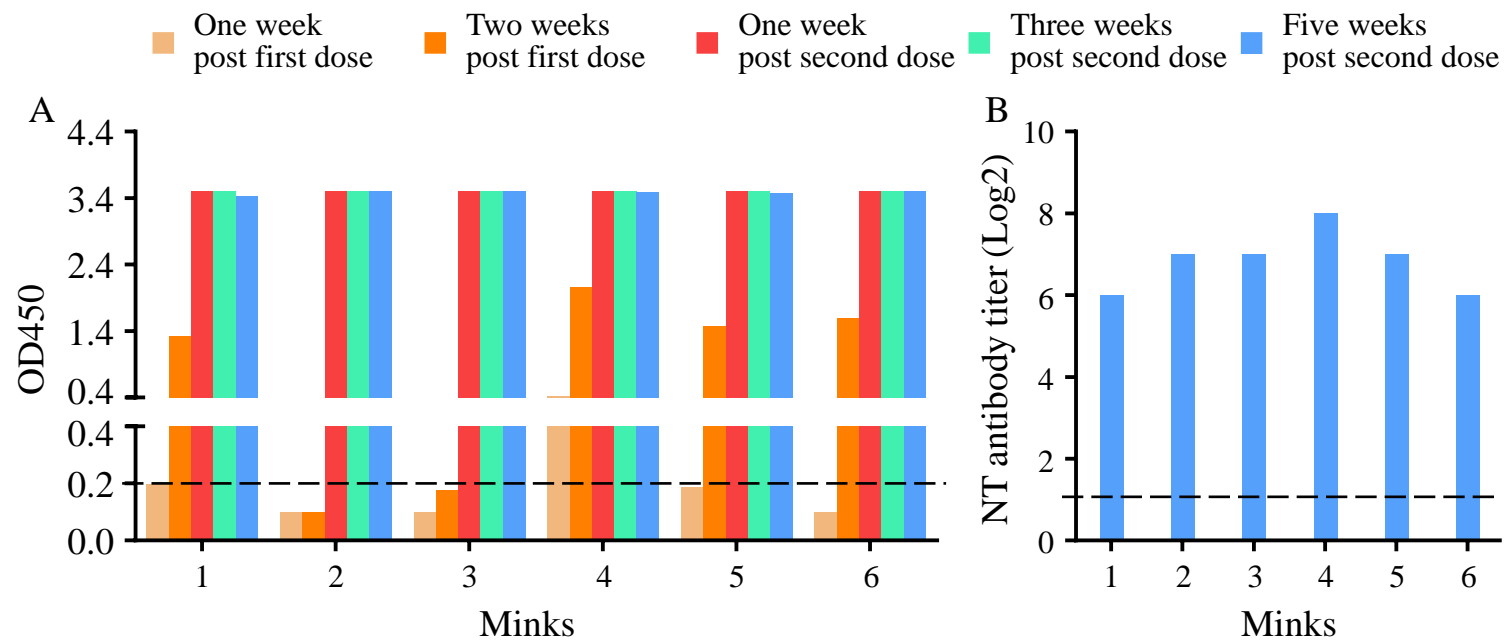

Supplement: nwaa291_Supplemental_File [file nwaa291_supplemental_file.zip › Figure_S3.pdf]
